# Production and Mechanical Characterization by Compression Tests of Al Alloys with Weaire–Phelan Cells Manufactured by the Lost-PLA Technique

**DOI:** 10.3390/ma18061261

**Published:** 2025-03-13

**Authors:** Alessandra Ceci, Corrado Cerini, Girolamo Costanza, Maria Elisa Tata

**Affiliations:** Industrial Engineering Department, University of Rome Tor Vergata, 00133 Rome, Italy; alessandra.ceci@uniroma2.it (A.C.); corrado_cerini@libero.it (C.C.); elisa.tata@uniroma2.it (M.E.T.)

**Keywords:** 3D cellular structures, Al foams, Weaire–Phelan, mechanical properties, compression behavior, experimental tests

## Abstract

In this study, the mechanical behavior of AA6082 foams with Weaire–Phelan (WP) cell structures under compressive loading was analyzed. The foams were produced using the lost-PLA replication method, a cost-effective and straightforward manufacturing technique. A total of six aluminum alloy samples were fabricated and subjected to compression tests to assess both their mechanical performance and the repeatability of the results. The produced foams demonstrated a well-defined morphology and high-quality surface finish, accurately replicating the geometries of the original PLA 3D-printed templates. The experimental density of the foams closely matched theoretical values, confirming the consistency of the replication process. The compressive stress–strain response of the Weaire–Phelan cell foams displayed an initial linear elastic region, followed by three distinct plateau regions with increasing stress levels. The final densification phase occurred when the structure could no longer accommodate further plastic deformation, leading to a sharp increase in the compression load. From the stress–strain data, the specific energy absorption of the foams was calculated. The average specific energy absorption was measured to be 4 J/cm^3^, with a standard deviation of 0.49 J/cm^3^ across the six tested samples. These results indicate reliable mechanical performance and reproducibility of the manufacturing process, making these foams suitable for applications requiring energy absorption and lightweight structural components.

## 1. Introduction

Cellular structures are receiving increasing attention in scientific and engineering research due to their exceptional mechanical [1,2,3,4], thermal [5,6,7], acoustic [8], and structural [9,10] properties. In particular, the demand for lightweight structures that ensure high mechanical strength and superior overall performance is continuously growing across many engineering disciplines [11,12]. To meet these seemingly conflicting requirements, precise control of material porosity is essential [13,14,15]. With recent advancements in Additive Manufacturing technologies, it has become possible to fabricate integrated components composed entirely or partially of cellular structures with optimized geometries [16,17]. The biomedical [18,19,20], civil engineering [21,22], aerospace [23,24], and transportation sectors [25]—where weight reduction is critical—have greatly benefited from the capability to produce free-form geometry components and high energy absorption [26,27] properties in static and dynamic conditions [28,29]. They are best effective also in terms of heat-transfer performance, showing a goodness factor that is 12% greater than Kelvin-cell and open-cell foam [30,31]. An interesting simulation of thermal transport in open-cell metal foams in periodic unit-cell structures is reported in [32].

Cellular structures can generally be divided into two broad categories: stochastic structures, such as foams, and non-stochastic structures, such as honeycombs or three-dimensional lattice materials. Among these, Weaire–Phelan structures represent an intriguing geometric configuration inspired by the minimization of surface energy in foam materials [33]. First introduced by Weaire and Phelan in 1994 [34], this structure is characterized by a periodic arrangement of non-equivalent cells that optimize space filling with a favorable volume-to-surface ratio. Potential applications include energy absorption, thermal and acoustic management, and structural uses in lightweight components [34,35,36].

Many research studies focus their attention on the mechanical characterization of Ti alloys (mainly Ti6Al4V) manufactured by additive manufacturing [37,38] and Ni [39]. The main goal of this work is to fill the gap regarding the compressive behavior of Weaire Phelan Al alloys manufactured by a simple and cost-effective technique. A numerical simulation for Al foams’ behavior [39,40,41] is an alternative solution to laboratory tests for reducing costs of material design.

An innovative method for producing such structures is the “lost-PLA” technique [17,42]. This approach, derived from traditional investment casting, combines 3D printing with thermoplastic materials, such as polylactic acid (PLA), with precision casting techniques to achieve complex geometries in metallic or ceramic materials. The process begins with the digital design and production by additive manufacturing of the PLA model, followed by plaster filling of the cavities, removal of the base material through combustion or dissolution, and finally, casting of the molten alloy. This method is particularly suitable for reproducing intricate structures like the Weaire–Phelan configuration, which would otherwise be challenging or impossible to fabricate using conventional techniques.

In this study, strategies for the design and manufacturing of Weaire–Phelan structures using the lost-PLA method, focusing on process optimization, and the mechanical characterization of the produced samples are described.

## 2. Materials and Methods

This study focuses on the fabrication of EN AW 6082 cellular structures using the lost-PLA technique. This method is derived from the ancient lost-wax casting process, adapted for modern applications through the use of 3D printing technology. The process begins with the creation of a 3D model of the desired structure using Solidworks 2020. Once the design is finalized, the model is exported in STL format, enabling the 3D printer to interpret and fabricate the design using PLA filaments.

Following the printing of the PLA model, the next step involves the creation of a negative mold to facilitate metal casting. To achieve this result, the printed model is coated with a slurry mixture of water and plaster. To ensure successful removal of the PLA during burnout and to allow molten metal to flow into the mold cavity, vent channels (sprues) are integrated into the design. These sprues are formed by attaching additional PLA elements to the model. After printing, these sprues serve as conduits to facilitate the removal of PLA and the subsequent introduction of molten metal.

**Figure 1:**
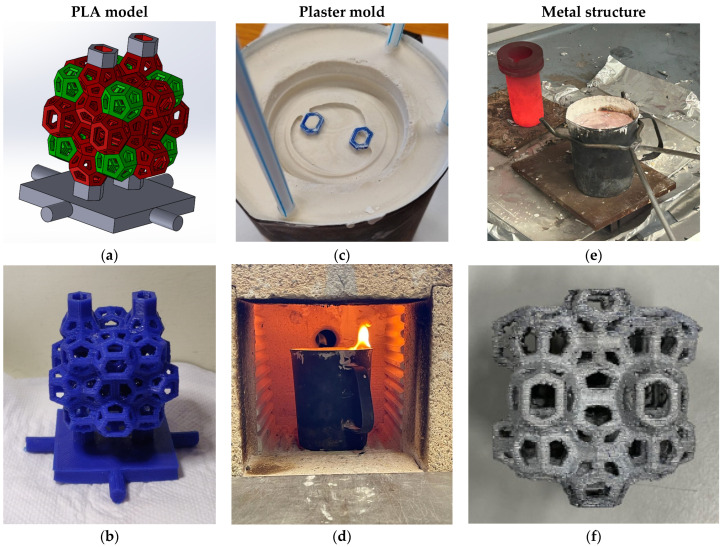
(**a**) CAD model; (**b**) 3D-printed PLA model; (**c**) plaster casting; (**d**) burnout; (**e**) gravity casting; (**f**) final AA 6082 structure.

The PLA model, complete with sprues, is placed within a stainless-steel container, which is then filled with liquid plaster. Once the plaster solidifies, the mold undergoes a drying process. Subsequently, the mold is subjected to thermal treatment in a furnace, where the PLA is burned out, leaving a cavity that precisely replicates the original design. The result is a detailed and accurate imprint suitable for metal casting. A schematic representation of this manufacturing process is illustrated in Figure 1a–f.

This technique offers significant flexibility and versatility, as CAD parameters can be precisely adjusted to produce a wide range of geometries. Furthermore, the method allows for accurate prediction of the surface area and volume of the final cellular structures prior to casting. In this study, a cellular structure composed of 33 Weaire–Phelan cells (24 tetrakaidecahedra and 9 dodecahedra) was selected for characterization. The number of cells was determined in order to completely fill the molds.

The lost-PLA process effectively enables the production of intricate and customizable cellular structures, making it suitable for applications that demand lightweight, high-strength materials.

### Weaire–Phelan Cell Selection

**Figure 2:**
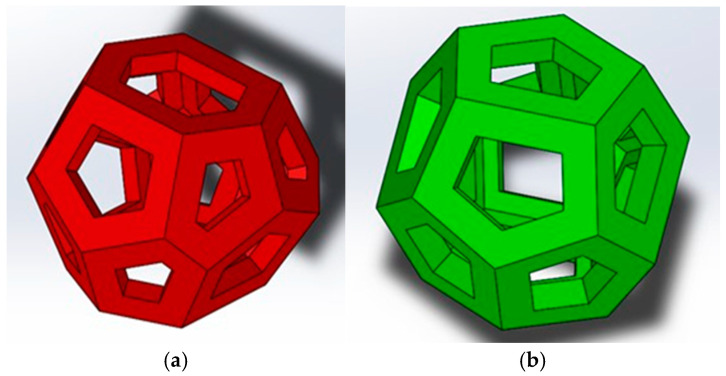
Weaire–Phelan cell: (**a**) hollow tetrakaidecahedron; (**b**) hollow dodecahedron.

**Figure 3:**
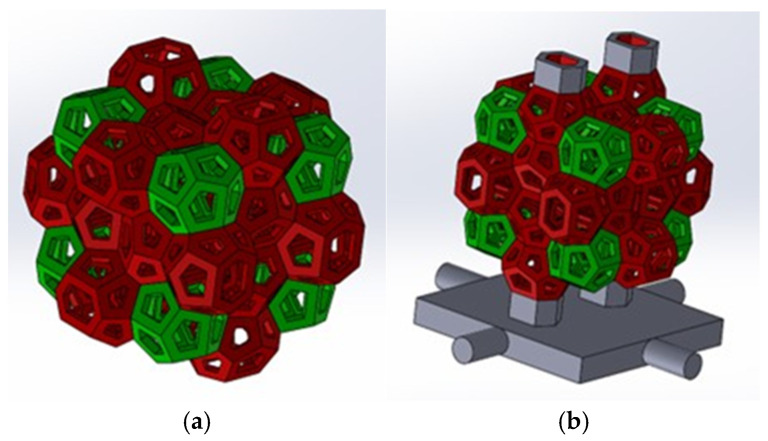
(**a**) Structure with Weaire–Phelan cells for compression tests; (**b**) structure with Weaire–Phelan cells for compression test with supports employed for casting phase.

The Weaire–Phelan structure is formed by the appropriate coupling of several tetrakaidecahedra (truncated hexagonal trapezoidal) and dodecahedra (Figure 2). In geometry, the Weaire–Phelan structure is a three-dimensional configuration that represents an idealized foam of equal-size cells, with two different shapes (Figure 3).

The parameters selected for sample printing are outlined below, with the corresponding quantities presented in Table 1:

- Speed: 40 mm/s (PLA) and 60 mm/s (PVA).
- Temperature: 200 °C (extruders) and 60 °C (printing plate).
- Layer thickness: 0.25 mm.
- Fill density (relative to bulk PLA): 20%.

The relative density (ρ*) of a cellular structure is defined as the ratio of the density of the cellular structure (ρ_cell_) to the density of the solid material (ρ_s_):

(1) $$\rho^{*}=\frac{\rho_{cell}}{\rho_{s}}=\frac{m_{cell}}{m_{s}}$$

As the densities are computed for the same unit volume, the relative density (ρ*) can be expressed as the ratio of the masses. Since the material remains constant, ρ* can also be represented as the ratio of the volumes. This approach allows for the straightforward calculation of the theoretical value of ρ* (Table 2), starting from volume and surface.

After printing, the PVA support is removed by immersing the sample in water at room temperature for approximately 5 h, followed by drying the PLA model. Once dried, straws are inserted, and a mixture of water and plaster is prepared inside the mold. In accordance with the plaster manufacturer’s instructions, a water-to-plaster ratio of approximately 40:100 was used. The mixture, once it reaches the appropriate consistency, is poured to cover the sample completely, leaving only the casting channels and straw vents exposed. After this, the mold is allowed to dry for at least 24 h to minimize the moisture content before placing the mold in an oven.

**Figure 4:**
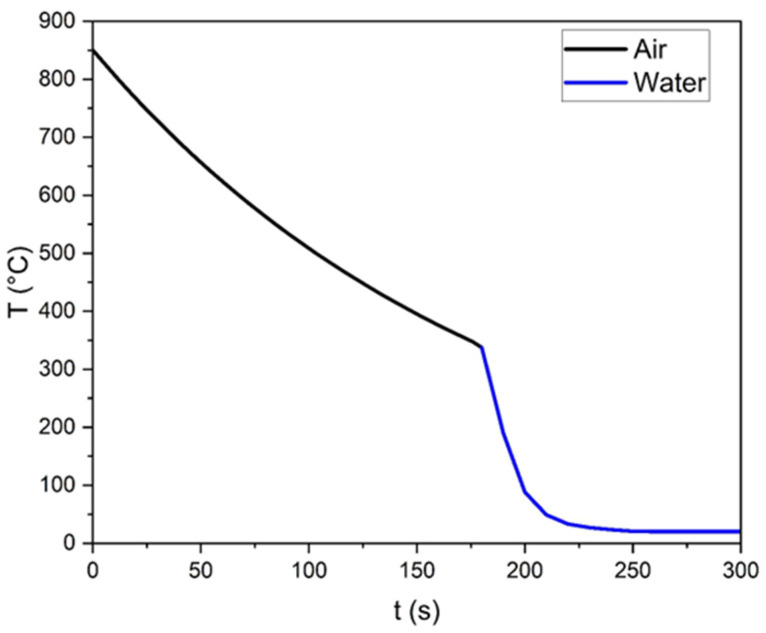
Temperature vs time profile of solidification and cooling process for a Weaire–Phelan AA6082 structure.

Once the plaster mold is completely dry, the oven, with the mold inside, is heated up to 900 °C, and the aluminum alloy billet (EN AW 6082) is heated up in a separate furnace set at 850 °C. When both furnaces reach the target temperatures, a type k thermocouple is placed in the plaster mold through one of the venting channels and casting begins. Till the alloy is in the molten state, vibrations are applied to the mold to promote air release and prevent clogging of the casting channels. Once solidification is completed, the sample is quickly cooled by immersion in water. At room temperature, the model is cleaned by removing excess plaster and by trimming the bases and allowances formed during the casting process. The temperature vs time profile of a typical solidification process is shown in Figure 4.

**Figure 5:**
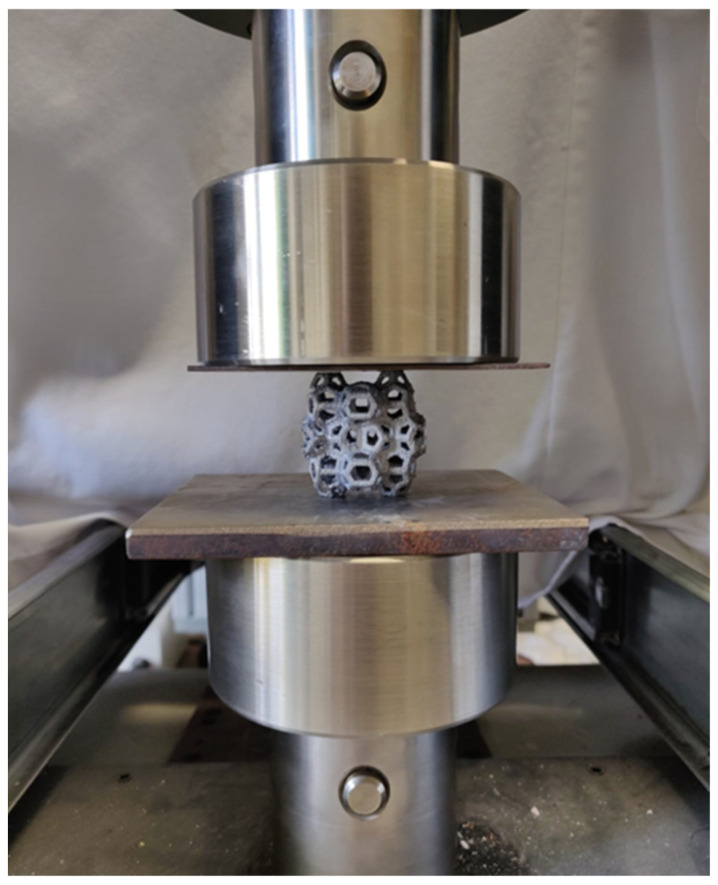
Compressive plates of the MTS tensile machine employed for the mechanical characterization of the manufactured samples.

All samples underwent compression testing using a 50 kN tensile-compression MTS Machine (Eden Prairie, MN, USA), with the testing parameters set to a crosshead speed of 6 mm/s and a maximum applied load of 48 kN (Figure 5). For each test, three samples were characterized, and the average values were considered.

The characterization of the microstructure of the AA 6082 alloy was performed through metallographic investigations. Samples taken from the WP manufactured structures were embedded in a bicomponent thermosetting resin, so that the polishing process with a grinding abrasive paper and diamond paste could be easily carried out. After polishing, the sections were chemically etched in a Keller solution (0.5% HF and distilled water) and were observed with a metallographic Leitz optical microscope and Hitachi S-2460N scanning electron microscope coupled with an energy dispersive X-ray analyzer.

**Figure 6:**
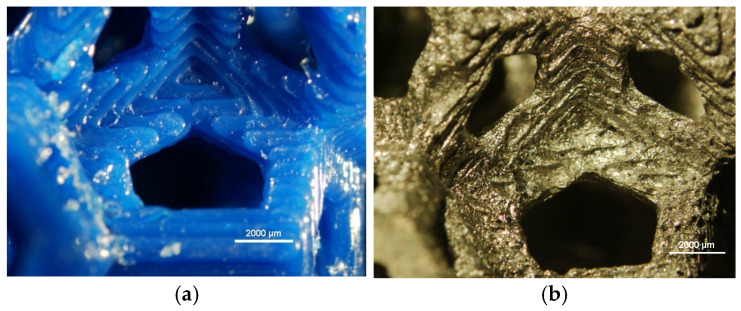
Details of the Weaire–Phelan manufactured structures. (**a**) Macrograph of Weaire–Phelan cells for PLA 3D-printed samples. (**b**) Macrograph of Weaire–Phelan cells for AA6082 cast samples.

The hardness of the as-cast AA 6082 alloy was analyzed by means of the Vickers hardness tester Shimadzu (HV 0.3) according to the standard ISO 6507-1:2023 [43] (Figure 6). Six measurements were performed on as-cast samples in slow cooling conditions (air solidified then water cooled with the WP structure). The hardness value was compared with that in the T6 conditions.

X-ray diffraction was carried out on as-cast samples that were representative of the AA 6082 solidified alloy. The Philips 1729 X-ray diffractometer was employed with Mo-Kα radiation (λ = 0.070930 nm) as the X-ray source and a θ−2θ goniometer. A scanning step of 0.05° (2θ) and a 5 s counting time for each step were set as the parameters.

## 3. Results and Discussion

The printed and cast samples were examined using a stereo microscope, and the images are reported in Figure 6. As observed in the images, the casting process accurately replicates the surface morphology and fine details.

**Figure 7:**
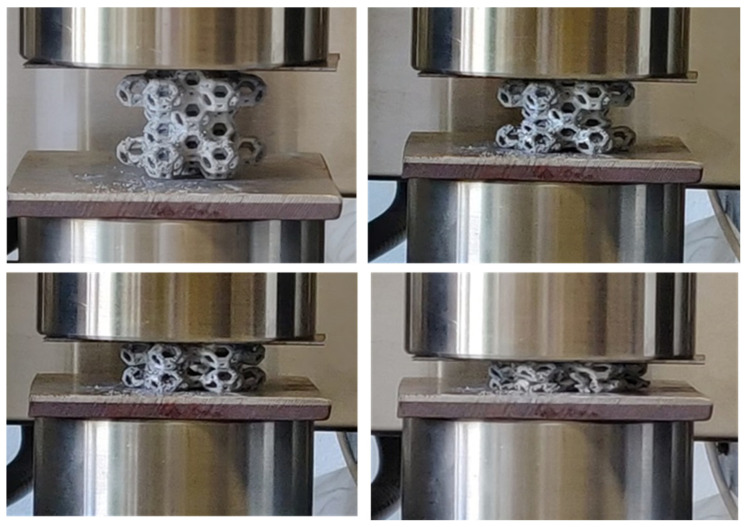
Successive compression steps of Al WP cell structures between the compression plates of the tensile-compression machine.

Six manufactured samples with the WP structure were subjected to compression testing in the as-cast conditions. Four WP samples (WP2, WP3, WP4, and WP6) were compressed in the same direction as the PLA prototypes were printed by the 3D printer, meaning that the layer planes were orthogonal to the applied load. The two samples (WP1 and WP5) with small casting defects were compressed laterally, with the layer planes parallel to the applied load. The sample dimensions and geometry are provided in Table 3. During the compression tests, load-displacement data were collected as raw data. Pictures taken at four successive compression steps are reported in Figure 7. These data were then processed by dividing the load by the original cross-sectional area and the displacement by the initial height of the foams, yielding the stress–strain curves for all samples.

**Figure 8:**
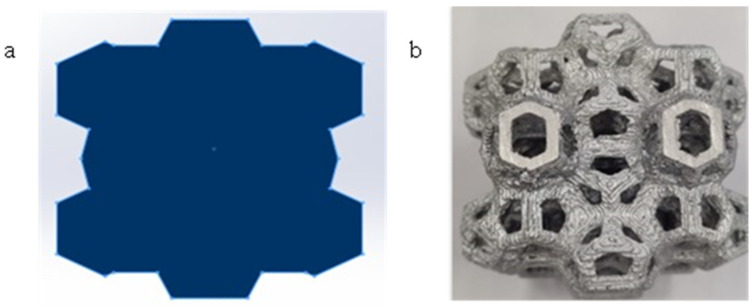
(**a**) Identification of the resistant section for Weaire–Phelan cell specimens; (**b**) top view of the manufactured AA6082 samples.

To get the σ-ε curves, the effective cross-sectional area of the samples needed to be determined. Since the geometry of the Weaire–Phelan cell specimens led to variations in the cross-sectional area during compression, an increased and standardized area was considered (Figure 8). This area, SR, corresponds to the one defined by the perimeter of the structure when viewed from above. By using this overestimated value for the cross-sectional area, the σ values and energy absorption were conservatively estimated.

Additionally, by incorporating the material properties into a CAD program, the theoretical weight of the structure after casting was calculated.

As shown in the data presented in Table 4, the printing and casting process introduced an average error of up to 14% in the final weight. The most successful filling was achieved for sample number 3, whereas samples number 1 and 5 were moderately damaged during the casting, resulting in the loss of part of the structure.

**Figure 9:**
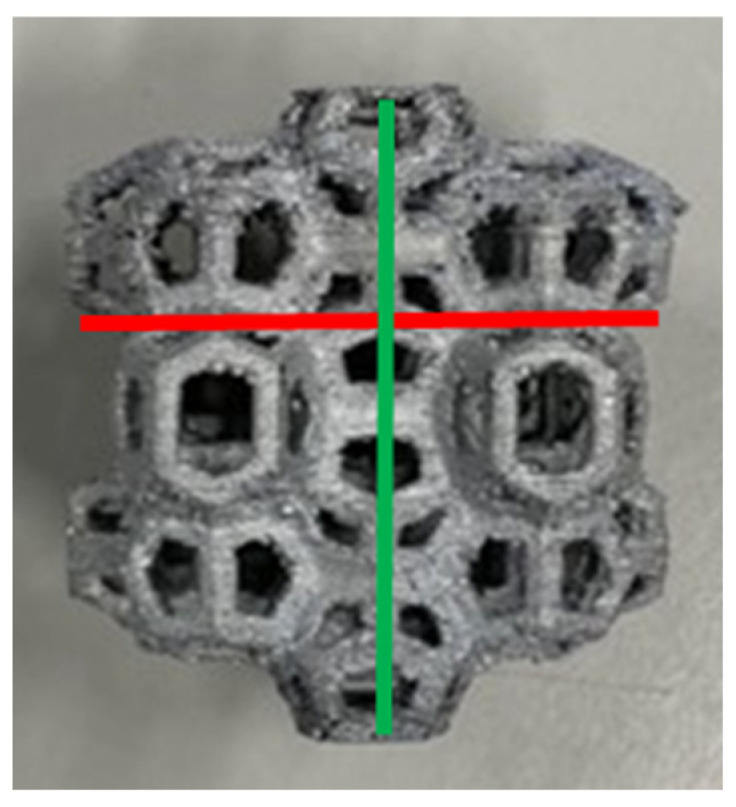
L_x_ (red) and L_y_ (green) identification for the Weaire–Phelan cell structures.

The following symbols are employed in the tables presented hereafter (Figure 9):

- L_x_ = maximum length of the specimen along x;
- L_y_ = maximum length of the specimen along y;
- H_i_ = initial height of the specimen;
- H_f_ = final height of the specimen;
- W_i_ = initial weight of the specimen;
- W_f_ = final weight of the specimen;
- S_R_ = resistant section.

The allowances and bases were removed using a cutting machine, which may account for the variations in the height values of the samples, as they are influenced by cutting precision. The geometric characteristics and weight of the samples are provided in Table 4, before the compression tests, and in Table 5, after the tests.

**Figure 10:**
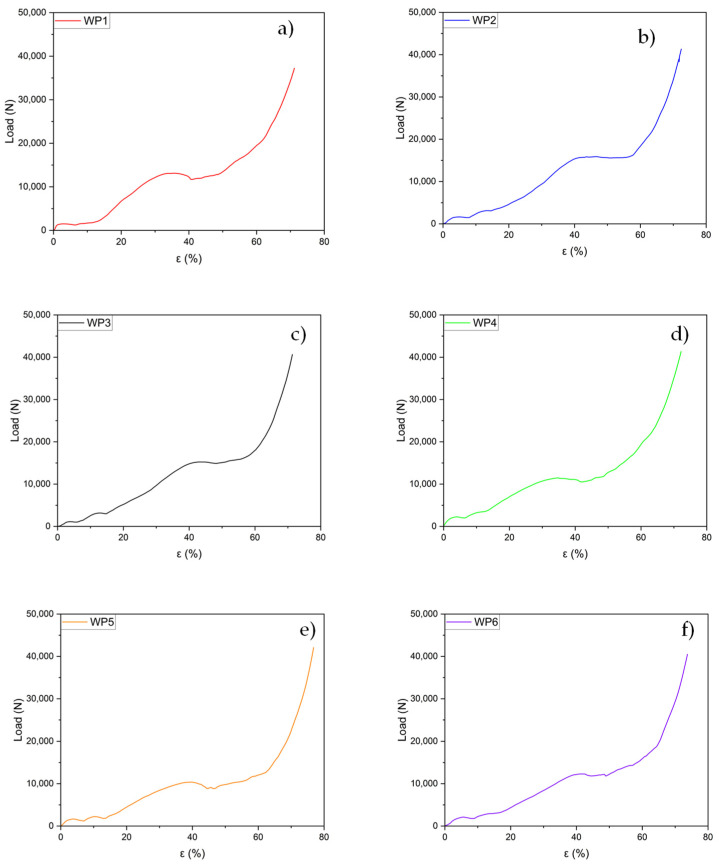
(**a**–**f**): Load–strain curves for six Weaire–Phelan cell structures.

**Figure 11:**
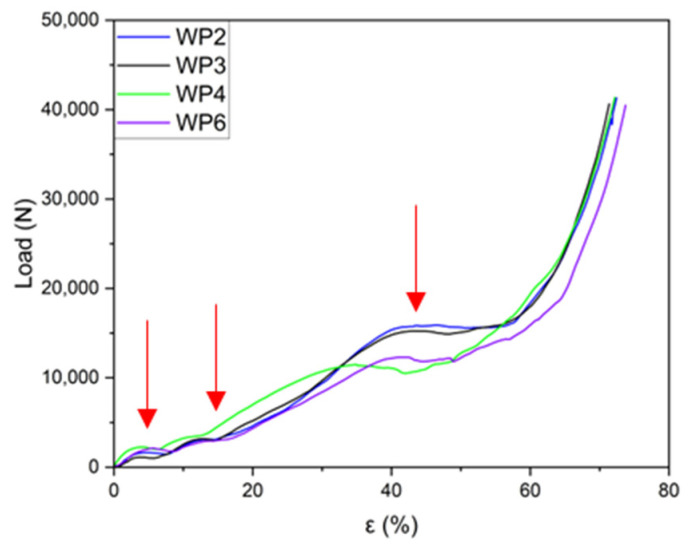
Load–strain curves for the samples (WP2, WP3, WP4, and WP6) were compressed in the same direction as the PLA prototypes were printed by the 3D printer. Plateau loads indicated by red arrows.

The load–strain curves from the compression tests for the six Weaire–Phelan cell structures are presented individually in Figure 10 a–f to highlight the detailed progression of them. All the fabricated samples display similar behaviors in their compression curves (Figure 10 and Figure 11). Following the initial linear region, a significant plateau-like phase is observed, where the load remains nearly constant or increases gradually. This plateau stage extends to approximately 65–70% of the total strain. Each curve exhibits varying degrees of load fluctuations (load drop), which can be attributed to the structural deformation and plastic collapse occurring at the weaker planes, after which the load increases again. A comprehensive comparison of all the curves is provided in Figure 11.

From Figure 10, it can be observed that the behavior of the various curves, compressed in the direction orthogonal to the layers, are quite similar. Three distinct plateaus at different load values can be identified, corresponding to the collapse of an entire plane of cells, which is influenced by the geometry of the produced structures. During the compression tests, it is evident that the collapse of the structure occurs gradually. Initially, the top and bottom layers, consisting of the two cells in contact with the plates, fail, showing the first plateau. Subsequently, the two intermediate layers, which are wider, collapse, leading to the second plateau. Finally, the central layer fails, resulting in the third plateau before the final densification stage.

**Figure 12:**
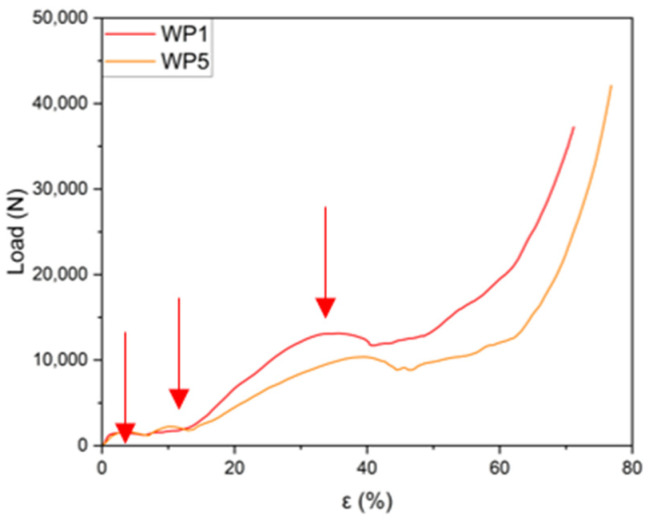
Load–strain curves for the samples (WP1 and WP5) compressed laterally, with the planes of the layers parallel to the applied load. (Plateau loads indicated by red arrows).

Although sample WP2 exhibited an incomplete structure, this does not seem to affect the curve’s behavior, as the graph closely overlaps with the curve of WP3, which was fully intact. The curve for sample WP4 is the only one that significantly deviates from the profiles of the other three under identical compression conditions. It appears to follow a trend more similar to the curves for samples WP1 and WP5, as shown in Figure 12, even though these were tested with the load applied parallel to the layer planes.

For the structures compressed parallel to the layer planes, Figure 12, the curves also exhibit similar trends. In this case, three distinct plateaus can also be identified before the final densification stage. However, the third plateau appears at lower strain values (35% instead of 43%, average values), which can be ascribable to both internal defects in the central part of the sample and the orientation of the layers relative to the applied load. The mechanical behavior is similar to that of Kelvin-cell Al structures [42], with three different plateaus instead of two, fluctuations of greater loads, and at the same time, higher energy absorption.

**Figure 13:**
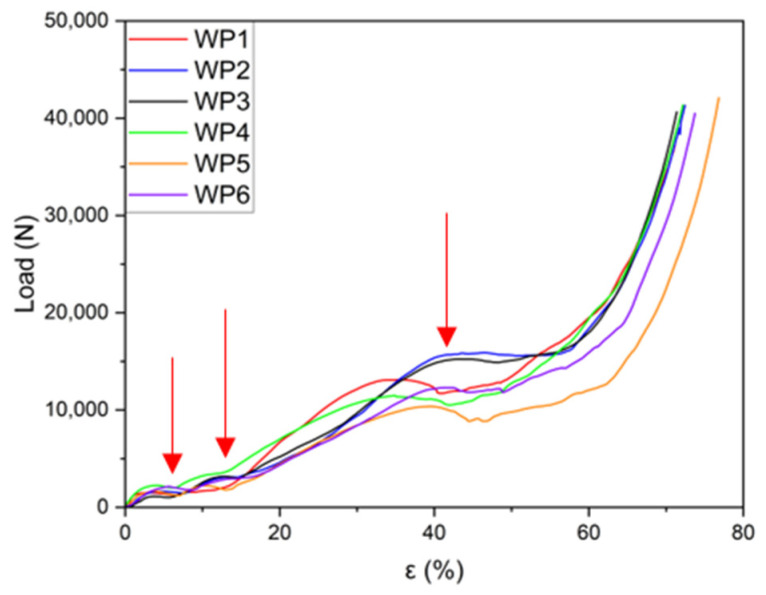
Load–strain curves (comparison) for the six manufactured Weaire–Phelan cell structures. Plateau loads are indicated by red arrows.

As shown in Figure 13, the load–strain graphs for the six manufactured samples demonstrate a high degree of repeatability. This indicates that except for minor load fluctuations caused by the progressive deformation of the structure, the material’s behavior is largely predictable. Following the initial linear region of the curve, a significant plateau phase is observed, extending up to an approximately 57% strain. Beyond this point, a sharp rise in load indicates the onset of final densification, which continues until a strain of about 70%.

The results of the compression tests are summarized in Table 6, specifically highlighting the first plateau load (L_p1_), the second plateau load (L_p2_), and the third plateau load (L_p3_). The corresponding strain values (ε_p1_, ε_p2_, and ε_p3_) are also provided in the same table.

The specific energy absorbed prior to final densification was determined by integrating the area under the stress–strain curve for a strain of up to 60% for all samples. The calculated results are summarized in Table 7, which includes the specific energy absorption for each individual sample, along with the average value and the standard deviation.

**Figure 14:**
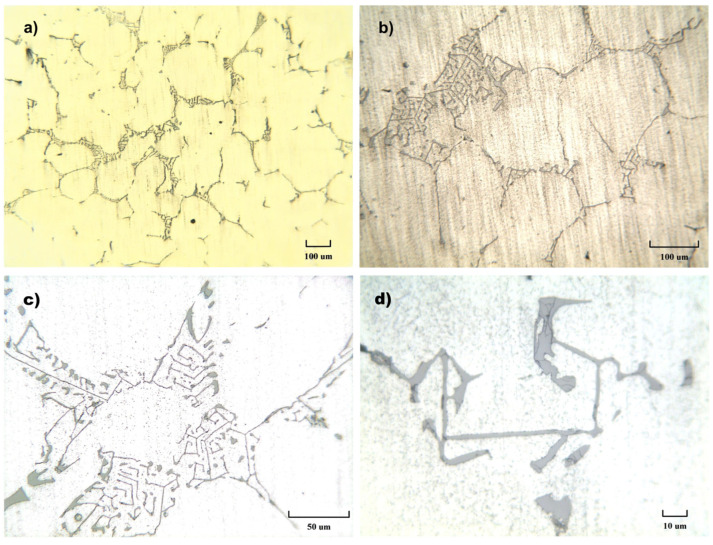
Microstructures of the AA 6082 alloy after the casting process at 100× (**a**), 200× (**b**), 500× (**c**), and 1000× (**d**).

**Figure 15:**
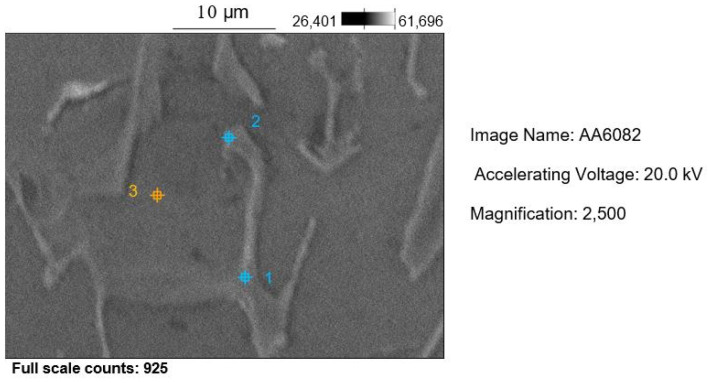
SEM micrograph of the AA 6082 alloy after the casting process (2500×).

The micrographs in Figure 14a–d show the microstructure of the Al 6082 alloy at different magnifications, ranging from 100× to 1000×: an interdendritic space of the α-Al solid solution and the precipitates of the intermetallic phases. The latter consist of Mg_2_Si, *β*-AlFeSi, and *α*-AlFeMnSi. The metallographic structures are in good agreement with the literature [44] on as-cast alloys. A SEM micrograph (2500×) is reported in Figure 15, and EDS analysis results on points 1–3 are reported in Table 8.

The microstructure and the corresponding chemical composition of the as-cast WP foam were evaluated with a scanning electron microscope with X-ray energy dispersive spectroscopy. In Figure 15, it is possible to observe two main regions: an AlFeMgMnSi-rich zone (point 1 and point 2) and a α-Al solid solution (point 3). Due to the slow cooling solidification condition (air cooling) and in the absence of age-hardening thermal treatment, there is no evidence of hardening precipitates as confirmed also from the obtained microhardness values.

Vickers hardness tests (HV0.3) were performed on AA 6082 T6 alloys and on the as-cast samples (air solidified then water cooled). The results (average values) and standard deviations are reported in Table 9. As expected, the highest hardness value (109.3 HV) was measured on the alloy in the T6 state (starting alloy). At the same time, the air-solidified sample exhibited the lowest hardness value (50.4 HV).

**Figure 16:**
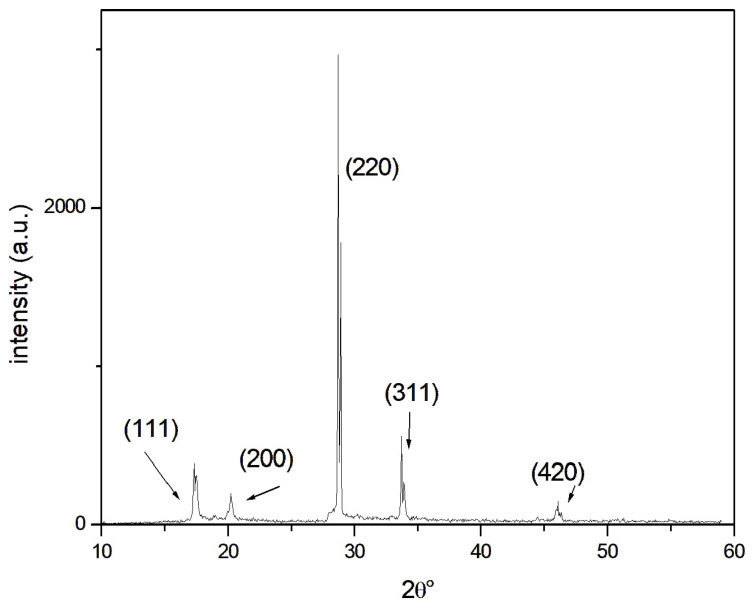
XRD pattern for the AA 6082 alloy after the casting process, collected employing Mo-kα radiation with a θ-2θ goniometer, a scanning step of 0.05° (2θ), and a counting time of 5 s. α-Al solid solution diffraction peaks were identified.

The X-ray diffraction pattern obtained for the air-cooled AA 6082 alloy (Figure 16) proves the main phase observed with optical microscopy (α-Al solid solution). The identified diffraction peaks on the spectrum exhibit high intensities and are well matched with JCPDS card n. 4–787. Small-size precipitates, locally identified (Figure 15 and Table 8) with EDS analysis, were not detectable with XRD due to their small size and the low volume content, in agreement with the thermal treatment the alloy underwent.

As evidenced above, a cheap and easy approach for producing a metal porous structure with a pre-determined design of the periodic cell (Weaire–Phelan) has been presented in this work. The proposed method allows us to manufacture Al lattice specimens with a good surface finishing (replicating the PLA-printed ones) with a high level of accuracy. When subjected to compression tests, the six manufactured samples showed good repeatability of the load–strain graphs. Four samples were compressed orthogonal to the layer planes, while two samples were compressed parallel to the layer planes. In both cases, except for small load fluctuations, the material’s behavior was homogeneous and largely predictable. After the initial linear stage of the curve, three plateaus were evidenced in the ranges of 2–5%, 8–11%, 40–45%, respectively, and can be associated with the successive strain of the structure at increasing stress levels. The results from compression tests are reported in Figure 13, as well as the plateau loads (L_p1_, L_p2_, and L_p3_) and strain levels in Table 6. The microstructure of the alloy in the as-cast condition is in good agreement with that well-known from the literature: an interdendritic space of the α-Al solid solution and the intermetallic phases Mg_2_Si, β-AlFeSi, and α-AlFeMnSi, as evidenced by optical microscopy and SEM observations. The measured hardness values of the AA 6082 alloy in the as-cast conditions (air solidified and successively water cooled) are in line with the expectations for those obtained by thermal treatment on microstructures (50.4 HV) as well as for the AA 6082 T6 base material (109.3 HV).

## 4. Conclusions

Weaire–Phelan cell AA 6082 structures were produced using the lost-PLA replication method, an economical and straightforward approach for creating metal porous structures. This process offers significant flexibility and versatility, allowing the desired parameters to be defined in a CAD program and facilitating the reproduction of a wide range of geometries. The mechanical behavior of these porous structures was characterized through static compression tests conducted on six samples of identical geometry. Based on the analysis of the resulting stress–strain curves, the following conclusions were drawn:

(1) Geometrical Consistency: The geometric characteristics and weight of the Weaire–Phelan cell structures demonstrate consistent repeatability across the manufacturing process. The theoretical geometric and physical values obtained from the CAD model align closely with the measured values.
(2) Compression Behavior: Static compression tests revealed the typical behavior of open-cell structures. The curves display an initial linear region, followed by three distinct plateaus where the planes progressively collapse as plastic deformation occurs. Final densification takes place when the structure reaches its deformation limit, resulting in a sharp increase in compressive load.
(3) Repeatability of Results: the stress–strain curves exhibit good repeatability, confirming that the manufacturing technique is reliable and that the production process is stable and reproducible.
(4) Specific Absorbed Energy: the specific absorbed energy values are consistently distributed among the six samples, with an average value of 4 J/cm^3^ and a standard deviation of less than 0.5 J/cm^3^.
(5) XRD, OM, SEM, and EDS analyses confirmed the microstructure (α-Al solid solution) deriving from the as-cast condition. The characteristic peaks on the spectrum represent high intensities and are well matched with JCPDS card n. 4-787. Small-size precipitates (AlFeMgMnSi-rich zone), locally identified with EDS analysis, are not detectable with XRD due to their small size and low volume content. The mechanical properties of the alloy, hardness in particular, are in good agreement with the cooling condition.

Future research should focus on a comparative study of the mechanical performance and energy absorption of Weaire–Phelan AA6082 cell structures and Kelvin cell structures, particularly under compressive loading conditions. The effects of ageing treatment on the mechanical properties as well as compression behavior should be analyzed too.

## Figures and Tables

**Table 1:** Material length (m) and weight (g) for printing samples.

Sample	PLA [m]	PVA [m]	Weight PLA [g]	Weight PVA [g]	Weight Tot [g]
Weaire–Phelan	2.59	4.05	20.5	31.7	52.2

**Table 2:** Ideal sizes obtained for the samples.

Sample	Volume V [mm^3^]	Surface S [mm^2^]	S/V [mm^−1^]	ρ* Theoretical	ρ* Theoretical [%]
Weaire–Phelan	9762.3	24,693.1	2.53	0.296	29.6

**Table 3:** Geometrical characteristics and weight for the AA 6082 Weaire–Phelan structures where ρ_0_ is the relative density of the Al 6082 alloy.

Samples	L_x_ [mm]	L_y_ [mm]	H_i_ [mm]	S_R_ [mm^2^]	W_i_ [g]	ρ/ρ_0_ (%)
WP1	39.6	39.1	39.3	1287.7	22.6	25.4
WP2	39.5	39.6	40.6		23.0	25.9
WP3	39.7	39.7	40.1		24.9	28.0
WP4	39.7	39.6	39.8		23.3	26.2
WP5	37.0	39.6	39.4		19.1	21.5
WP6	39.5	39.5	41.2		22.6	25.4

**Table 4:** Weight of the Weaire–Phelan cellular structures.

Samples	Weight [g]	Weight CAD [g]	ΔW [g]	ΔW [%]	$\bar{\Delta W}\;[\%]$	ρ/ρ_0_
WP1	22.6	26.3	3.7	14	14	25.4
WP2	23.0		3.3	12		25.9
WP3	24.9		1.4	5		28.0
WP4	23.3		3.0	11		26.2
WP5	19.1		7.2	27		21.5
WP6	22.6		3.7	14		25.4

**Table 5:** Characteristic sizes for Weaire–Phelan samples after the compression tests.

Samples	H_f_ [mm]	ΔH [mm]	ε [%]	$\bar{\varepsilon }$ [%]	W_f_ [g]	ΔW [g]	$\bar{\Delta W}$ [g]
WP1	12.0	−26.3	−67	−70	22.3	−0.3	−0.1
WP2	12.3	−28.3	−70		22.9	−0.1	
WP3	12.6	−27.5	−68		24.8	−0.1	
WP4	12.0	−27.8	−70		23.2	−0.1	
WP5	10.3	−29.1	−74		18.9	−0.2	
WP6	11.7	−29.5	−72		22.6	−0.0	

**Table 6:** Plateau load values in compression tests for WP structures.

Samples	L_p1_ [kN]	ε_p1_ (%)	L_p2_ [kN]	ε_p2_ (%)	L_p3_ [kN]	ε_p3_ (%)
WP1	1.54	1.6–6.7	1.67	7.3–12.5	12.8	32.4–48.9
WP2	1.67	2.5–8.2	3.09	11.2–14.8	15.84	40.9–57.1
WP3	1.16	2.7–5.7	3.09	11.8–14.6	15.20	40.3–55.7
WP4	2.19	2.4–6.2	3.60	8.1–12.3	11.46	32.4–47.6
WP5	1.67	2.3–6.1	2.19	8.6–12.3	10.30	39.9–56.0
WP6	2.06	3.3–7.5	2.96	10.4–15.8	12.23	39.3–49.7

**Table 7:** Specific absorbed energy in Weaire–Phelan cell structures.

	WP1	WP2	WP3	WP4	WP5	WP6
*E_spec_* [J/cm^3^]	4.34	4.35	4.32	4.18	3.15	3.69
$\bar{E_{spec}}$ [J/cm^3^]	4.00					
Dev stand	0.49					

**Table 8:** EDS analysis results points 1–3 in Figure 15 (wt %).

	Al	Mg	Fe	Cu	Si	Mn
Point 1	90.8	0.8	3.2	0.3	2.7	2.2
Point 2	92.4	0.6	2.6	0.2	2.4	1.8
Point 3	99.2	0.2	0.1	0	0.4	0.1

**Table 9:** HV0.3 for AA 6082 cast samples.

Samples	HV	Standard Deviation
As-cast (Air solidified then water cooled)	50.4	3.6
AA 6082 T6	109.3	1.8

## Data Availability

The original contributions presented in this study are included in the article. Further inquiries can be directed to the corresponding author.
